# Treatment of gastric cancer peritoneal metastases: role of cytoreductive surgery and hyperthermic intraperitoneal chemotherapy

**DOI:** 10.1093/bjs/znae149

**Published:** 2024-07-02

**Authors:** Andreas Brandl, Johanna W van Sandick

**Affiliations:** Department of General, Visceral and Transplantation Surgery, University Hospital Heidelberg, Heidelberg, Germany; Department of Surgical Oncology, The Netherlands Cancer Institute, Amsterdam, The Netherlands

## Background

Gastric cancer (GC) is the fifth most common malignancy in the world, with an incidence of approximately 1 million new patients worldwide in 2022^1^. Due to its aggressive behaviour and the late appearance of symptoms, such as bleeding, loss of appetite, weight loss, and abdominal pain, most patients have locally advanced and/or metastatic disease at first presentation. Especially in patients with diffuse-type GC, the peritoneum is the most common site of metastatic spread. At least 40% of patients with GC are confronted with peritoneal metastases (PM) during their disease, leaving them with a miserable prognosis of a median overall survival of 7–9 months.

Following European Society for Medical Oncology (‘ESMO’) and National Comprehensive Cancer Network (‘NCCN’) guidelines, the treatment of choice for patients with metastatic GC is two-agent systemic chemotherapy, preferably a platinum/fluoropyrimidine doublet, in combination with targeted therapy or immunotherapy considering human epidermal growth factor receptor 2 (HER2) and programmed death-ligand 1 (PD-L1) status^2,3^.

## History and status of intraperitoneal chemotherapy

Through continuous scientific efforts that aim to improve the treatment and prognosis of patients with GC, multimodal therapeutic concepts have been created and evaluated. The backbone of anticancer therapy is systemic chemotherapy, which has become more effective in recent years. This was partially due to the discovery of new therapeutic targets, such as HER2 and PD-L1, depending on the various expressed antigens. Given the high frequency of PM, the obvious area to target is the intra-abdominal cavity.

A potential benefit of intraperitoneal chemotherapy in combination with cytoreductive surgery (CRS) in patients with GC and PM has been demonstrated in several cohort studies. In a large retrospective multicentre study from France including 277 patients, the median overall survival was 18.8 months after CRS and hyperthermic intraperitoneal chemotherapy (HIPEC) *versus* 12.1 months after CRS alone^4^. In a large series from Japan including 419 patients who underwent neoadjuvant intraperitoneal chemotherapy followed by surgery, the median overall survival was 20.5 months in patients in whom a complete cytoreduction (CC-0) was achieved^5^.

The recently published randomized controlled GASTRIPEC-I trial was unable to confirm this survival advantage (median overall survival of 14.9 months after CRS/HIPEC *versus* 14.9 months after CRS alone; HR 0.72), although better progression-free survival and other distant metastasis-free survival were found for CRS/HIPEC *versus* CRS alone^6^. Randomization was performed before systemic treatment, which led to a dropout rate of 55 of 105 included patients, mostly because of tumour progression. Overall survival was calculated from the time of randomization, that is around 2–3 months before CRS/HIPEC (not—as in previous studies—from the day of CRS/HIPEC until last follow-up). The GASTRIPEC-I trial illustrated the real-world difficulties of successfully treating patients using a multimodal concept. Because many patients were excluded from the per-protocol analysis, determining which patients benefitted from the intensive multimodal approach used in this trial was not possible.

After completion of the dose-finding phase I–II PERISCOPE I trial, which proved the feasibility of gastrectomy combined with CRS/HIPEC using 460 mg/m^2^ hyperthermic oxaliplatin and 50 mg/m^2^ normothermic docetaxel, the randomized Dutch PERISCOPE II trial was initiated to investigate the additional value of CRS/HIPEC compared with systemic treatment alone. Eligible patients have resectable GC, a peritoneal cancer index (‘PCI’) of less than seven and/or tumour-positive cytology, and an absence of disease progression during systemic chemotherapy^7,8^. The results of this study are highly anticipated as they will close the gap between surgical trials evaluating CRS/HIPEC and historical medical oncological trials, wherein the extent of PM was not known or inclusion was not restricted to patients with PM only. Inclusion in the PERISCOPE II study is hampered by strict patient selection, patient preferences, and the inability to open new centres during Clinical Trials Information System transition, such that an extension of the study towards 2026 is anticipated.

An important alternative strategy is to focus on the prevention of metachronous PM in patients operated on for GC, based on the motto ‘An ounce of prevention is worth a pound of cure’. The French GASTRICHIP trial and the German PREVENT trial are currently investigating the use of prophylactic HIPEC (30 min of 250 mg/m^2^ oxaliplatin in the GASTRICHIP trial and 90 min of 75 mg/m^2^ cisplatin in the PREVENT trial) to reduce the risk of metachronous PM^9,10^. The primary endpoints were chosen to be 5-year overall survival and progression-free survival respectively and therefore the results of the studies are expected in 2026.

## Molecular treatment challenges and future perspectives

In the field of molecular targeted therapy for the treatment of PM in GC, several challenges are encountered due to the complex nature of this disease. The first feature to overcome is its heterogeneity. GC is characterized by significant molecular and genetic heterogeneity, both within primary tumours and between primary tumours and metastases. This leads to variability in treatment response and the emergence of resistant clones, making it difficult to develop effective targeted therapies.

Another so far unresolved difficulty is the tumour microenvironment. In GC, it is characterized by immune suppression, angiogenesis, and stromal interactions, which can contribute to treatment resistance. Strategies aimed at modulating the tumour microenvironment, such as immune checkpoint inhibitors, have shown promise, but are not universally effective.

Third, there is the issue of acquired resistance. After an initial response to chemotherapy or targeted therapies, the development of acquired resistance is common in metastatic GC. Resistance mechanisms may involve secondary mutations, activation of alternative signalling pathways, or adaptive changes in the tumour microenvironment, necessitating the development of innovative combination therapies to overcome resistance. This underlines the importance of prevention or a first complete tumour control.

Precision medicine is certainly one of the major goals in the future. While improving the understanding of the molecular landscape of GC, personalized approaches reign supreme.

It includes adequate patient selection for intensive therapies, as well as ‘do no further harm’ to those who will not benefit. This clinical decision-making requires sophisticated methods of tumour-response prediction and assessment. In this regard, liquid biopsies hold promise in providing real-time information on tumour evolution. In addition, the role of repeated intraperitoneal chemotherapy *versus* a single shot in CRS/HIPEC deserves further investigation^11^.

In the upcoming years, results from RCTs will reveal the role of CRS/HIPEC in GC patients. At this point in time, for the individual patient with GC and PM, and no disease progression during systemic treatment, who asks for surgery and HIPEC, counselling is essential, based on the available data. Collaborative efforts between researchers and clinicians are crucial to advance the field and improve outcomes for patients with this aggressive disease.
